# HETEROGENOUS EFFECTS OF MEDICARE BENEFIT FOR ADVANCE CARE PLANNING CONVERSATION BY SOCIODEMOGRAPHIC FACTORS

**DOI:** 10.1093/geroni/igad104.2366

**Published:** 2023-12-21

**Authors:** Yifan Lou

**Affiliations:** Yale University, New Haven, Connecticut, United States

## Abstract

**Background:**

Although Medicare makes the option of physician-patient advance care planning (ACP) discussion available to everyone starting in 2016, older adults from disadvantaged backgrounds may be less likely to benefit from the policy. Motivated by Fundamental Cause Theory, this study examines the heterogenous policy effect, as a natural experiment, on older adults’ ACP uptake.

**Methods:**

Data were from Health and Retirement Study 2012-2020 with 15,647 individuals. I used two quasi-experimental models – interrupted time series (ITS) analysis and difference-in-difference (DID) – to study ACP outcomes before and after the policy implementation between older adults who were Medicare-eligible and those who weren’t. I studied the heterogenous effects in stratified samples by race/ethnicity, immigration background, and socioeconomic status. I used coarsened exact matching to address the potential bias that the comparison group is not the ideal counterfactual control unit.

**Results:**

DID reveals that, in the total population, Medicare expansion was associated with a significant increase (0.05 percentage point) in the proportion of older adults who completed advance directives, but not for having informal conversations. Furthermore, I find no evidence that the Medicare expansion was effective in improving the rates of ACP among Black, Hispanic, Asian or Native American, or non-US-born older adults. Lastly, based on ITS results, the policy only has significant immediate policy effects (level change) on ACP uptake, but not for sustained effects (slope change). Discussions: The current policy reproduced the disparities in ACP. I discuss health policy recommendations to improve the ACP rate among older adults with disadvantaged backgrounds.

